# Supplementary material for: IL-6 trans-Signaling Impairs Sprouting Angiogenesis by Inhibiting Migration, Proliferation and Tube Formation of Human Endothelial Cells
Source: Cells. 2020 Jun 5;9(6):1414. doi: 10.3390/cells9061414 (PMC7349366; doi:10.3390/cells9061414)
Supplement: Supplementary file 1 [file cells-09-01414-s001.pdf]

Supplementary data

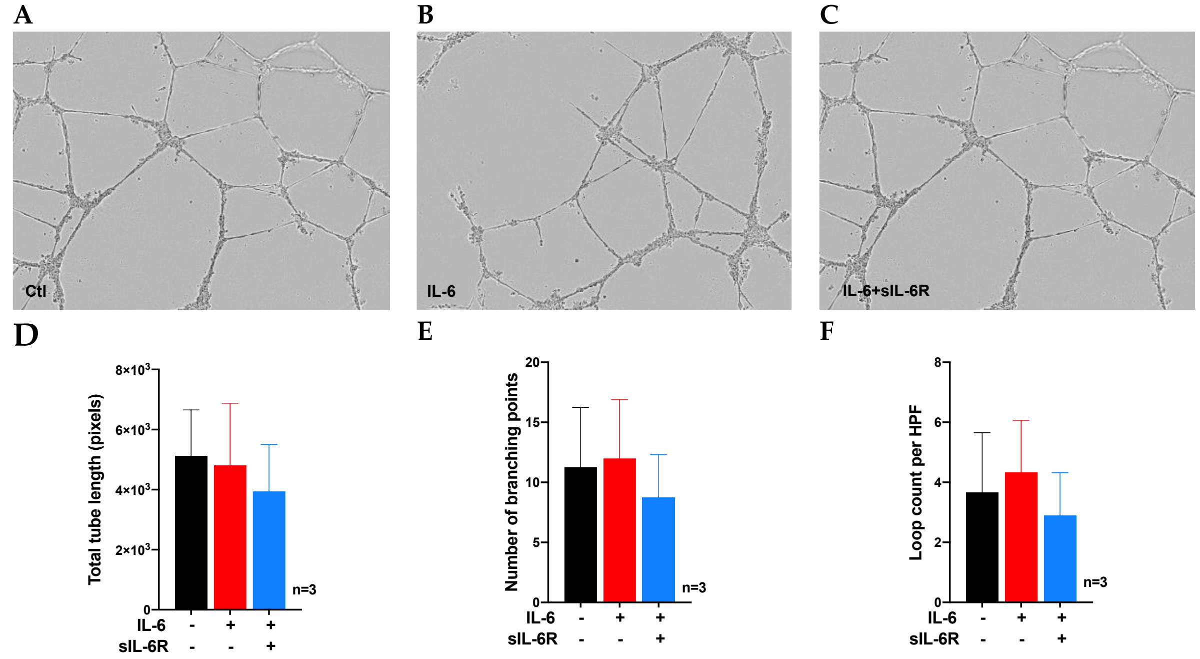

Supplementary figure 1. The effect of IL-6 signaling in tube formation of vascular ECs when added to the Matrigel at the same time as the cells. Representative images from tube formation of **(A)** unstimulated ECs or **(B)** ECs treated with IL-6 alone or **(C)** in combination with sIL-6R. Quantification of the tube formation is presented as **(D)** total tube length, **(E)** number of branching points and loop count **(F)**. Data is presented as mean ± SEM of 3 experiments each run-in duplicate.

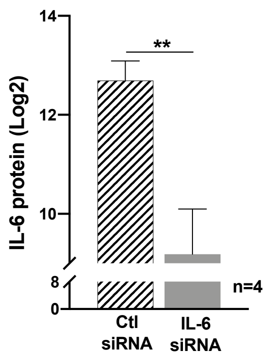

Supplementary figure 2. Olink normalized protein expression (NPX) data on the expression of IL-6 after knockdown using stealth siRNAs. Data is presented as mean ± SEM of 4 experiments each run-in duplicate. **p < 0.01 compared to control.

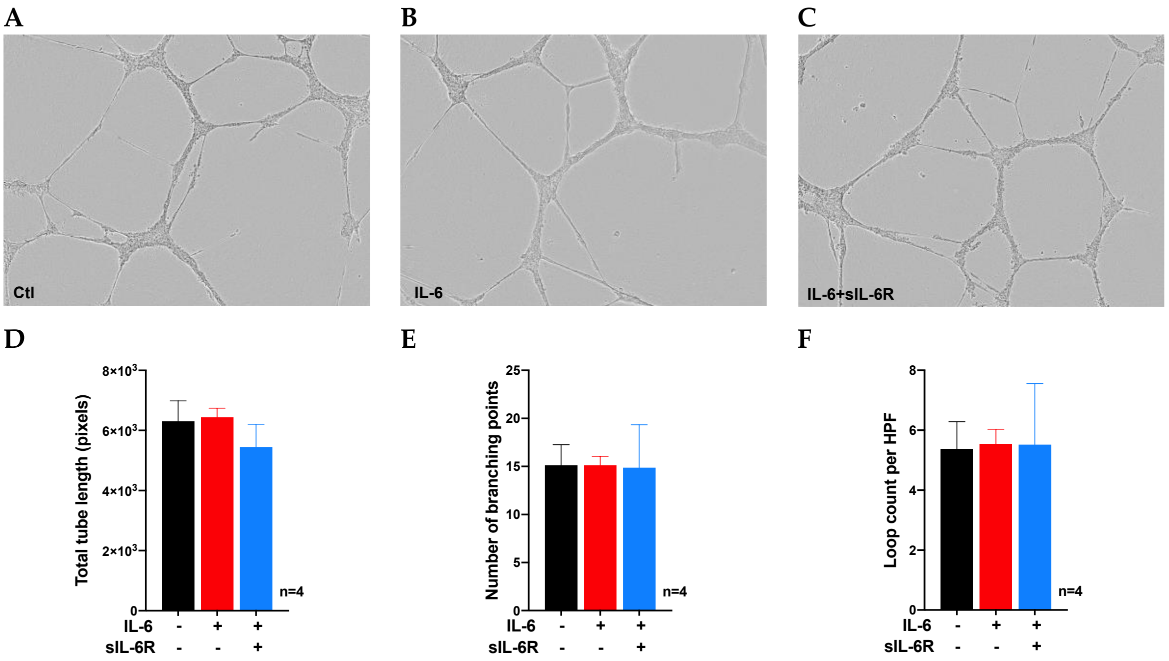

Supplementary figure 3. Tube formation of vascular ECs which have been pre-treated with IL-6 alone or in combination with sIL-6R on growth-factor-reduced (GFR) Matrigel. Representative images from tube formation of **(A)** unstimulated ECs or **(B)** ECs treated with IL-6 alone or **(C)** in combination with sIL-6R. Quantification of the tube formation is presented as **(D)** total tube length, **(E)** number of branching points and loop count **(F)**. Data is presented as mean ± SEM of 4 experiments each run-in duplicate.

Supplementary table 1. List of genes with Assay IDs in the angiogenesis array and respective ΔΔCt values, p-values and BH-p-values.

**Assay ID**	Target Name	ΔΔCt values	P-value	BH p-value
Hs00171042_m1	*CXCL10*	6,433	0,004	0,031
Hs00171467_m1	*SERPINF1*	5,929	0,020	0,065
Hs00300159_m1	*HGF*	5,002	0,003	0,031
Hs00236077_m1	*CEACAM1*	4,723	0,020	0,065
Hs00383235_m1	*PTN*	4,386	0,045	0,100
Hs00601975_m1	*CXCL2*	4,309	0,009	0,048
Hs00171022_m1	*CXCL12*	3,887	0,001	0,022
Hs00998026_m1	*PDGFRA*	3,401	0,012	0,052
Hs00427220_g1	*PF4*	2,885	0,022	0,065
Hs00900373_m1	*CHGA*	2,292	0,190	0,278
Hs00559786_m1	*ANGPTL1*	2,181	0,001	0,018
Hs01001469_m1	*ITGB3*	2,116	0,004	0,031
Hs00196470_m1	*ENPP2*	1,934	0,000	0,018
Hs00913333_m1	*TNNI1*	1,911	0,328	0,442
Hs00272659_m1	*LYVE1*	1,886	0,101	0,175
Hs00157317_m1	*TYMP*	1,140	0,060	0,126
Hs01105174_m1	*BAI1*	0,776	0,024	0,066
Hs01077958_s1	*IFNB1*	0,716	0,089	0,158
Hs00896294_m1	*PROX1*	0,685	0,018	0,063
Hs01922614_s1	*S1PR1*	0,651	0,162	0,253
Hs00199608_m1	*ADAMTS1*	0,582	0,133	0,218
Hs00176096_m1	*TEK*	0,544	0,017	0,063
Hs02379000_s1	*ANG*	0,431	0,102	0,175
Hs00187290_m1	*NRP2*	0,411	0,210	0,301
Hs00234042_m1	*PDGFB*	0,410	0,356	0,464
Hs00223332_m1	*TNMD*	0,392	0,735	0,793
Hs00765775_m1	*ANGPTL2*	0,388	0,138	0,222
Hs01568063_m1	*THBS2*	0,370	0,329	0,442
Hs00265254_m1	*FGF1*	0,263	0,068	0,132
Hs00170014_m1	*CTGF*	0,217	0,370	0,472
Hs00900054_m1	*VEGFA*	0,121	0,515	0,611
Hs00963711_g1	*GRN*	0,051	0,785	0,826
Hs00962914_m1	*THBS1*	0,049	0,922	0,945
Hs00171064_m1	*MDK*	0,040	0,627	0,714
Hs01011995_g1	*F2*	0,024	0,958	0,970
Hs01101127_m1	*ANGPTL4*	-0,015	0,970	0,970
Hs00826128_m1	*NRP1*	-0,065	0,732	0,793
Hs00176573_m1	*FLT1*	-0,118	0,163	0,253
Hs00611096_m1	*AMOT*	-0,130	0,381	0,473
Hs00176676_m1	*KDR*	-0,132	0,374	0,472
Hs00169777_m1	*PECAM1*	-0,133	0,646	0,725
Hs00181613_m1	*ANGPT1*	-0,143	0,750	0,798
Hs00166654_m1	*SERPINC1*	-0,199	0,280	0,389
Hs00234278_m1	*TIMP2*	-0,204	0,039	0,095
Hs00266645_m1	*FGF2*	-0,222	0,522	0,611
Hs00264877_m1	*PLG*	-0,245	0,895	0,929
Hs00233808_m1	*ITGAV*	-0,260	0,386	0,473
Hs00173634_m1	*VEGFB*	-0,282	0,044	0,100
Hs00993254_m1	*LECT1*	-0,356	0,663	0,735
Hs00153458_m1	*VEGFC*	-0,377	0,357	0,464
Hs00169867_m1	*ANGPT2*	-0,411	0,170	0,253
Hs00168433_m1	*ITGA4*	-0,423	0,083	0,154
Hs01549940_m1	*FN1*	-0,427	0,003	0,031
Hs00174344_m1	*CDH5*	-0,517	0,008	0,044
Hs00246256_m1	*FST*	-0,562	0,169	0,253
Hs00168405_m1	*IL12A*	-0,563	0,035	0,086
Hs00178500_m1	*TIE1*	-0,579	0,005	0,031
Hs01047677_m1	*FLT4*	-0,609	0,055	0,120

Hs00194179_m1	*HSPG2*	-0,648	0,015	0,060
Hs00165949_m1	*TIMP3*	-0,654	0,010	0,049
Hs00197064_m1	*FBLN5*	-0,667	0,022	0,065
Hs00189521_m1	*FIGF*	-0,676	0,123	0,206
Hs00208609_m1	*VASH1*	-0,703	0,021	0,065
Hs01098873_m1	*COL4A2*	-0,750	0,089	0,158
Hs00153304_m1	*CD44*	-0,868	0,066	0,132
Hs00266237_m1	*COL4A1*	-0,913	0,064	0,131
Hs00362096_m1	*EPHB2*	-0,936	0,016	0,063
Hs00181017_m1	*COL18A1*	-0,972	0,004	0,031
Hs00234422_m1	*MMP2*	-0,992	0,024	0,066
Hs99999918_m1	*TGFB1*	-1,016	0,001	0,022
Hs00188273_m1	*SEMA3F*	-1,151	0,030	0,081
Hs00270951_s1	*FOXC2*	-1,177	0,008	0,046
Hs00174781_m1	*EDIL3*	-1,248	0,003	0,031
Hs00266332_m1	*COL15A1*	-1,321	0,275	0,389
Hs00387364_m1	*PDGFRB*	-1,360	0,395	0,476
Hs00184728_m1	*SERPINB5*	-1,382	0,555	0,641
Hs99999083_m1	*CSF3*	-2,283	0,012	0,052
Hs00608187_m1	*TGFA*	-2,622	0,082	0,154
Hs00232618_m1	*HEY1*	-3,120	0,001	0,018
Hs00270802_s1	*TNFSF15*	-3,353	0,033	0,085
Hs00174103_m1	*IL8*	-3,457	0,042	0,099
Hs00174029_m1	*KIT*	-5,242	0,007	0,044
Hs99999901_s1	*18s*	HKG		
Hs99999905_m1	*GAPDH*	HKG_used for analyses		
Hs99999909_m1	*HPRT1*	HKG		
Hs99999908_m1	*GUSB*	HKG		
Hs00241027_m1	*FGA*	Not expressed		
Hs00168730_m1	*PRL*	Not expressed		
Hs00173564_m1	*FGF4*	Not expressed		
Hs00174877_m1	*LEP*	Not expressed		
Hs00260905_m1	*PROK1*	Not expressed		
Hs00174128_m1	*TNF*	Not expressed		
Hs00174143_m1	*IFNG*	Not expressed		
Hs00205581_m1	*ANGPTL3*	Not expressed		
Hs01022527_m1	*COL4A3*	Not expressed		
Hs00211115_m1	*ANGPT4*	Not expressed		
